# Supplementary figures and images for: Environmental justice and power plant emissions in the Regional Greenhouse Gas Initiative states
Source: PLoS One. 2022 Jul 20;17(7):e0271026. doi: 10.1371/journal.pone.0271026 (PMC9299318; doi:10.1371/journal.pone.0271026)

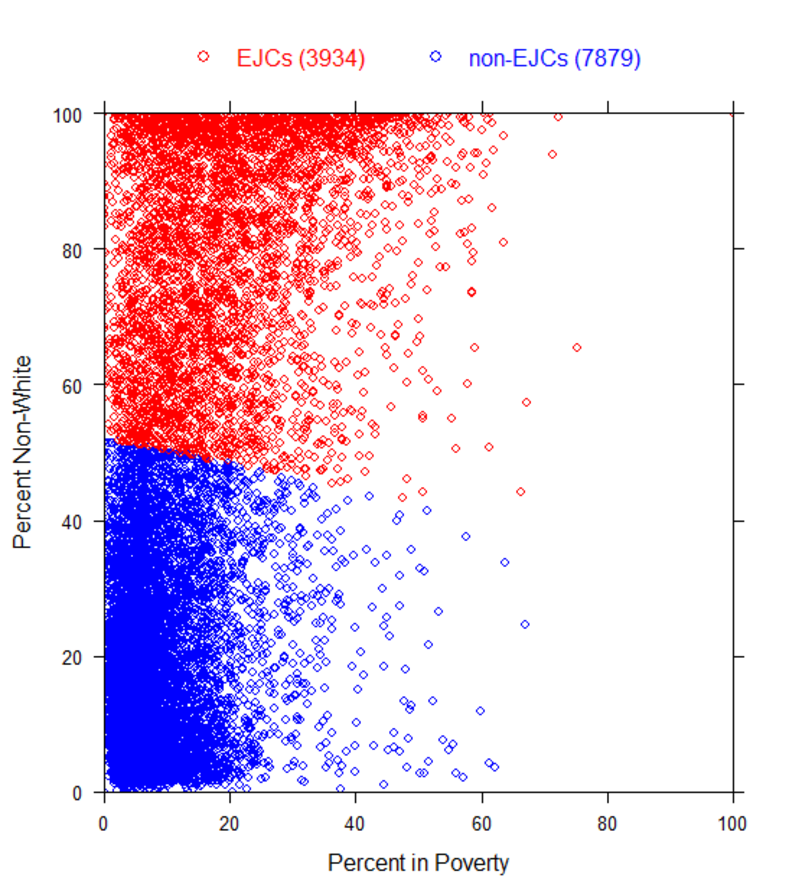

Supplement: S1 Fig — (TIF) [file pone.0271026.s001.tif]
