# Supplementary material for: Environmental justice and power plant emissions in the Regional Greenhouse Gas Initiative states
Source: PLoS One. 2022 Jul 20;17(7):e0271026. doi: 10.1371/journal.pone.0271026 (PMC9299318; doi:10.1371/journal.pone.0271026)
Supplement: Attachment — Submitted filename: comments_authors_RGGI_EJ.docx [file pone.0271026.s003.docx]

Review for PLOS ONE: “Exploratory Analysis of Changing Power Plant Pollution Burdens on Environmental Justice communities in the Regional Greenhouse Gas Initiative states.”

January 5, 2022

Comments for Authors:

This study addresses a vital question: whether market-based greenhouse gas emission reduction mechanisms such as RGGI increase environmental injustice. But it suffers from a fatal flaw in methodology and also from a lack of effort to explain the patterning of findings. The authors selected on the dependent variable (by only including the 285 Census tracts hosting one EGU, rather than all Census tracts in the 11 RGGI states). This has biased the analysis, which only compares host communities with host communities; non-host communities (that tend to be whiter and higher in income) are entirely left out of the analysis which results in under-measuring environmental inequality (see Mohai 1995, his famous critique of Anderton, Anderson, Oakes and Fraser 1994).

The authors demonstrate a good working knowledge of environmental justice issues; if they choose to redo the analysis to eliminate bias, more could be done to increase this study’s contribution to the scientific literature. Specifically, the paper suffers due to lack of explanation of the findings: what is driving the shift? The authors would need to at least try to explain the patterning of environmental inequalities found so that the paper can be stronger scientifically and also be a better contribution for crafting policy.

Comments on Introduction and Literature Review:

1. There needs to be some explanation of why RGGI matters for environmental inequality: how is RGGI hypothesized to decrease or increase environmental burdens for EJCs? How, if at all, might RGGI change the market calculation of what type of power plant might be profitable? The authors cite a few papers on these questions but I know there are many more.

Comments on data and variables:

1. It was not clear why “prime movers” mattered for this analysis. Do they have an effect on emissions? If so, how? If prime mover is just another way of referring to fuel, the authors should omit that phrase.

2. On Page 10-11, the authors don’t provide a clear definition of “environmental justice community.” Does this mean census tracts above a certain threshold of percent people of color AND above a certain threshold of people in poverty (or was it OR a certain threshold of people in poverty)? And what were “non-EJC outliers?”

3. On page 21, describing the limitations of the study, the authors state that it would have been preferable to consider people living under the poverty level (presumably, for a comparison with those living at poverty level), but due to limitations in Census data this was not possible. Given that the poverty level is such an outmoded undercount of people living in poverty, I doubt that comparing people in poverty with people in deep poverty would have been as useful as comparing people in poverty with those better off. But the authors could have compared people at 100% of poverty level with people at 200% of poverty level (the near-poor). These data are available in Table POV01, which should be available for the years up to 2014.

Comments on methods:

1. Unfortunately, the methods used in the analysis were fatally flawed: the authors selected on the dependent variable by only including the 285 census tracts that host EGUs in the analysis. This has biased the analysis because it compares host communities with host communities, leaving non-host communities entirely out of the analysis. The scope of the analysis is very important, as this methodological choice has been proven to underestimate environmental inequality (see Mohai 1995).

Aside from this serious problem, other methodological choices were not adequately explained:

2. It is not clear why the distance of four miles was chosen to represent proximity to EGUs. Is there some scientific justification for defining proximity in terms of four miles from the centroid of each census tract, rather than one mile, two miles, etc.?

3. The authors seemed to miss out on some of the precision with which distance-based measures can find environmental inequalities. Did people of color or people in poverty *live closer* to EGUs than others? Or was a four-mile radius the basis for the analysis?

Comments on interpretation of findings:

1. The authors should at least try to find explanations for these patterns, such as the finding that there were fewer coal-fired EGUs and more natural gas-fueled EGUs in EJ communities. Below are some possible explanations that could be explored:

- Natural-gas power plants may be newer than coal-fired power plants, and thus may have been sited recently. Since 1990 there has been more opposition to siting environmentally hazardous facilities; non-EJ communities may have successfully blocked new power plants, leaving EJ communities “the path of least resistance.”
- This also could be driven by zoning: it is well established that minorities and the poor are more numerous in places zoned for industrial use.
- Investment in new (natural gas) power plants or in the conversion of outmoded ones may have occurred in more densely populated urban and metropolitan areas versus rural areas. Populations in rural areas tend to be whiter and poorer.
- It is likely that there is a lot of variation in the patterns of proximity to EGUs in RGGI-participating states: states that are white, rural and poor with little investment in new infrastructure (e.g., Maine) may be the same states with more outmoded coal-fired plants; states that are urban and diverse with extremes of wealth and poverty (e.g., New Jersey) may have more natural gas plants.
- If any of these explanations are correct, they might explain the finding that CO2 and SO2 emissions in EJCs were higher between 1995-2005 but reductions were larger in EJCs after 2007.

2. The finding regarding emissions appears to be a bright spot: emissions have gotten lower since 2005 for EJCs. But the authors needs to do more to illuminate an understanding of this finding: did the pattern of emissions change due to the shift from gas to coal? Was it due to plant modernization (better pollution controls, cleaner fuels, or both)? Was it because methane emissions increased while CO2 and SO2 decreased? All of these questions are really crucial ones for redressing environmental inequalities.

3. Finally, what is the significance of the timing of the shift? Could it be due to policy (Energy Policy Act, Clean Power Plan, etc.)? Or the economy (gas prices)? If finding answers is beyond the scope of this study, the authors could speculate on these questions and suggest future research.

Comments on Conclusions:

1. The authors should mention the impacts on EJCs from the expansion of shale gas and oil, including fracking wells, oil trains, gas pipelines, liquid natural gas export terminals and compressor stations, among others.

Comments on Figures:

Figure 2: I was unable to understand from the manuscript why “prime mover type” was important. If it was because it affects the amount or toxicity of emissions, this should be made clear. I am not sure what this figure adds; if space limitations become an issue, I recommend omitting it.

Figure 3: what are the “outliers”?

Figures 5 – 10: There are too many figures, which take up space the authors need.

Mohai, Paul. "The demographics of dumping revisited: examining the impact of alternate methodologies in environmental justice research." *Virginia Environmental Law Journal* (1995): 615-653.
