# Supplementary material for: Environmental justice and power plant emissions in the Regional Greenhouse Gas Initiative states
Source: PLoS One. 2022 Jul 20;17(7):e0271026. doi: 10.1371/journal.pone.0271026 (PMC9299318; doi:10.1371/journal.pone.0271026)
Supplement: Attachment — Submitted filename: response to reviewers.docx [file pone.0271026.s004.docx]

**Reviewer #1**: - More suitable title should be selected for the article. Title should decrease to 10-12 words.

We have renamed our article to “Environmental justice and power plants in the Regional Greenhouse Gas Initiative states”.

**- The abstract should state briefly the purpose of the research, the principal results and major conclusions. An abstract is often presented separately from the article, so it must be able to stand alone.**

We agree with the reviewer that the abstract should clearly state the purpose, results, and major conclusions of the research. The purpose of our research is clearly stated in our abstract in lines 29-32: “In this study we address research gaps in historical and current ambient air emissions burdens in environmental justice communities from power plants participating in the Regional Greenhouse Gases Initiative (RGGI), the country’s first market-based power sector emissions reduction program.”

The principal results of our research are clearly stated in lines 32-37 (do note that we modified the language on the distance to power plants finding and added more precise information, as requested by reviewers):

*“We find that in RGGI states the percentage of people of color that live within 0-6 miles from power plants is up to 23.5 percent higher than the percent of the white population that lives within those same distance bands, and the percentage of people living in poverty that live within 0-5 miles from power plants is up to 15.3 percent higher than the percent of populations not living in poverty within those same distance bands. More importantly, the transition from coal to natural gas underway before RGGI formally started resulted in large increases in both the number of electric-generating units burning natural gas and total net generation from natural gas in environmental justice communities hosting electric-generating units, compared to other communities.”*

The major conclusions of our work are clearly stated in lines 39-43: “Our findings indicate that power sector carbon mitigation policies’ focus on aggregate emissions reductions have largely benefitted non-environmental justice communities and have not redressed the fundamental problem of disparities in pollutant burdens between EJ and non-EJ communities. These must be directly addressed in climate change and carbon emissions mitigation policy.”

**- It is suggested to present the structure of the article at the end of the introduction.**

We have added in lines 159-169 a paragraph describing the structure of our article.

**- The major defect of this study is the debate or Argument is not clear stated in the introduction session. Hence, the contribution is weak in this manuscript. I would suggest the author to enhance your theoretical discussion and arrives your debate or argument.**

We appreciate the reviewer’s suggestion to clearly state the problem we are addressing. In lines 49-142, we have clearly stated the need for our research, which is that in the US, climate change mitigation policy in the energy sector has not addressed environmental justice issues. Starting on line 84 (Electricity Sector Emissions and Environmental Justice section), we discuss the burden of mortality/morbidity attributable to electric generation, the lack of focus of such policies on the distribution of health and other benefits among environmental justice communities, and the potential for exacerbation of historical inequities that environmental justice scholars and advocates have voiced, if those concerns are unaddressed in climate change mitigation policy in the energy sector.

**- It is suggested to compare the results of the present research with some similar studies which is done before.**

We appreciate the suggestion to compare our study to other similar studies. As we have already noted in lines 448-474, the only other similar study to our knowledge is Cushing et al 2018, and we have compared our study to theirs.

**- More suitable title should be selected for the figure 4 instead of “Demographics in k-means cluster analysis of Census Tracts hosting EGUs in RGGI.”.**

We have modified what is now labeled Figure 4’s caption to “Percent people of color and percent in poverty statistics in k-means cluster analysis of Census Tracts hosting EGUs in RGGI.”.

**- It is suggested to add articles entitled “Okeke et al. City as Habitat; Assembling the Fragile City”, “Gibergans-Baguena et al. The Quality of Urban Air in Barcelona: A New Approach Applying Compositional Data Analysis Methods” and “Angelevska et al. Urban Air Quality Guidance Based on Measures Categorization in Road Transport” to the literature review.**

We have reviewed the suggested literature (Angelevksa et al. 2021; Okeke et al 2020; J. Gibergans-Báguena et al. 2020). While these papers provide valuable contributions to the literature on vehicular emissions, they are outside of the scope of our research centered on the environmental justice implications of power plant emissions in the US. Therefore, we respectfully decline to add these references to our literature review.

**- Page 11: the following paragraph is unclear, so please reorganize that:
“In addition, others have voiced more fundamental problems with carbon trading that do not center on emissions reductions, namely that carbon markets enable commodification of air pollution (30), effectively giving polluting facilities private property rights over the atmosphere.”**

We have rewritten the sentence to use clearer language:

*“In addition, others have voiced more fundamental problems with carbon trading, namely that carbon markets undermine efforts to decarbonize the global economy.”*

- DOI of the references must be added (you can use “https://crossref.org/").

We are using the PLOS ONE citation style as implemented in the Zotero reference system (MS Word plug-in). While our references database contains DOIs for references that have been assigned DOIs, the PLOS ONE citation style as implemented in the Zotero reference system does not add them to the references list. We will work with the PLOS ONE copy editor staff to add DOIs once the manuscript is accepted for publication.

**- Much more explanations and interpretations must be added for the Results, which are not enough.**

In response to this reviewer and other reviewers’ similar comments on broadening the discussion of our results, we have contextualized our findings more fully with regards to carbon reduction policies and achieving environmental justice in the discussion and conclusions sections.

**- Please make sure your conclusions' section underscore the scientific value added of your paper, and/or the applicability of your findings/results, as indicated previously. Please revise your conclusion part into more details. Basically, you should enhance your contributions, limitations, underscore the scientific value added of your paper, and/or the applicability of your findings/results and future study in this session.**

In our discussion, we have noted in lines 429-431 that the scientific value add of our paper is that our analysis constitutes the first systematic and empirical attempt to assess disparities in pollutant potential from electricity production between EJ communities and non-EJ communities in RGGI. In our conclusions, we discuss in lines 609-610 that our research can be useful to policymakers to directly consider the equity implications of policy options.

In terms of future research, we have noted that our findings on disparities between EJCs and non-EJCs leave many open questions for future research both to address inequities and to better understand that multiple factors that drive changes in power plant operations, siting, and emissions (lines 567-583).

**Reviewer #2**: Line 39: you need to define EJ for the ease of understanding before using it.

We have added a definition of environmental justice in lines 99-102.

**Lines 88-93: There is a repetition of a sentence. Please remove it.**

We have removed the repeated sentence, as it was a typographical error.

**Line 138: You have defined EJCs so you can use it here rather than using the complete name. Otherwise, there is no point of defining it earlier.**

As recommended, we have used the acronyms EJC and non-EJC throughout the manuscript when referring to the classification of Census Tracts in our analysis, including captions on all relevant tables and figures. One notable exception is that we have retained the use of the full terms in the Discussion in order to increase readability.
 **For population proximity, I would recommend generating a map of proximity using all the power plants to show the spatial heterogeneities in census tracts rather summarizing them into a line graph. Similarly, the maximum number for distance in Figure is 10 miles. Does this implies that no population/community is present beyond 10 miles proximity to power plants? I think a map is required to explain such ambiguity.**

As recommended, we have generated a map of proximity of census tracts to the nearest power plant in our analysis. We show the map below. We do not believe this map is a suitable substitution to the population proximity figure since it does not illustrate our finding that a larger fraction of EJ populations in RGGI states live closer to EGUs than the reference non-EJ populations. We instead propose to improve the existing figure. We realize that the 10-mile distance cutoff has generated confusion about the range of distances of the nearest power plant to census tracts. 10 miles was an arbitrary cutoff point that we selected in order to better illustrate the differences in the four lines; the maximum distance we found in our analysis is 53.8 miles. Therefore, we have generated a new Figure 2 (a two-panel figure now) showing the full range of census tract distance to power plants and a curve showing the difference between White/not-
White population and separately for in poverty/not in poverty populations (we have also submitted as an updated Figure 2 on this resubmission).

Figure: Distance of census tracts to the nearest power plant in our analysis

Figure: Revised Figure 2 showing full range of distance from EGUs in RGGI states

**For the identification of EJ communities, more details are required to clearly state what EJ and Non-EJ community in this study means. Is an EJ community with larger ethnic composition and poverty? or is it the opposite. Please clearly define these communities.**

We thank the reviewer for pointing out the lack of clarity in defining EJ communities. In the analysis section (“Identification of Environmental Justice Communities”, starting on line 237), we have reorganized the description of the methods for identifying EJ communities and added more detail to clearly communicate how we used the k-means cluster analysis to classify census tracts as EJCs or non-EJCs.
**For polluting potential of EGUs, it is strongly recommended to also check the significance of increasing or decreasing trends between 1995-2015 in the context of RGGI.**

We considered this question in an early, exploratory analysis in the initial stages of our research. We thought it would be important to assess trends in polluting potential before and after RGGI was implemented (2009). We split our dataset into a pre-RGGI period (1995-2009) and post-RGGI (2010-2015) subsets and conducted breakpoint analyses to look at the trends in the polluting potential indicators. We found that the explanatory power of our analysis was low in the pre-RGGI period likely in part because there were no data points for years 2001 and 2002. In addition, we also came to the conclusion that many other factors exogenous to RGGI, such as the ones we discuss in our conclusions, could account for changes in polluting potential in 1995-2015. We thus discarded this method in favor of the analysis we have presented, and have elevated the potential for RGGI-exogenous factors to account for the inequities we found.

**Lines 305-309: If the data for 2001 and 2002 is not used, how can one determined if the communities saw he "Sharpest" increase?**

*We realize the confusion that this can cause since there were no data points for years 2001 and 2002.Thus. we have modified the sentence to read (emphasis in “after” only here and not in the submission):*

*The number of EGUs in each type of community also grew during the time period: from 50 to 70 (a 40.0% increase) in EJ communities and from 193 to 225 (a 16.5% increase) in non-EJ communities, and both communities saw the sharpest increases* ***after*** *2003.*

**Line 316: Generation of what? Please clarify for the sake of readers**

Through our manuscript we refer to generation as electricity generation. However, to make this clearer, we have added “electricity” as a qualifier to the *Polluting potential of EGUs* subsection of the *Analysis* section and the Generation subsection (line 365, now renamed Electricity Generation).
**Line 370: there is a typo**

We have corrected this typographical error.

**In discussion, authors state that one of the key insights from this study is that EJ populations live closer to electric power plants. From where the authors deduce this insight? is it from Figure 3? if yes, the figure fails to explain which curves among the four are EJs and which ones are non-EJs.**

We realize that the language used does not clearly convey our finding that the cumulative population fraction of EJ populations is higher than that of non-EJ populations at any given distance from power plants. To clearly specify the population subgroups we analyzed in our proximity analysis, we have added the following sentence to the Analysis section (*Population proximity to power plants*):

*For the purposes of assessing population proximity to power plants, we deemed people of color and the population below the poverty threshold as environmental justice populations, and whites and the population at or above the poverty threshold as reference, non-environmental justice populations.*

In addition, we have modified the language in the Results section to better communicate our finding (see reply to the reviewer’s comment below on “threshold of ‘closer’”.

**Similarly, Authors describe that validation of inequity that low income and people of color continue residing closer to pollution emitting facilities. In Figure 3, 40% cumulative population for all four curves is at a distance of ~4 mile and the rest of the population is beyond this.**

We respectfully disagree with Reviewer 2’s statement that “40% cumulative population for all four curves is at a distance of ~4 mile”. We have reproduced Figure 2 below (showing the full range of distance as requested by Reviewer 2 above), indicating with a colored circle the cumulative population at 4 miles distance for each of the four cumulative population curves. This clearly shows that at a distance of 4 miles, the cumulative population percentage of both people of color and people living in poverty is higher than that for whites and people living with incomes above the poverty threshold. In our Results section we have used 4 miles as an example to illustrate the visual pattern shown in Figure 2 and have added language that makes this explicit (see next response on a related point raised by Reviewer 2).

**How the threshold of "closer" is defined in this study? and How to decide if 4 miles proximity is having health impacts? Similarly, did the authors use the temporal data on population (1995-2015) as well? if yes, you need to mention it. If not, how is it deduced that populations are still living closer to these facilities even after decades (line 389). There might be a decrease in the population residing the proximity of these facilities if one look at the temporal trends of population. However, it might require precise gridded population data to evaluate it. In a nutshell, the proximity section in this manuscript needs further explanations to clarify the issues.**

In the Results section, subsection *Population proximity to power plants*, we have used the term “closer” to describe the proximity of EJ and non-EJ populations to power plants. From Reviewer 2’s comments, we now realize that the sentence inaccurately describes the proximity differences we found in our cumulative population analysis.

We have rewritten this subsection to better communicate our findings. We have modified the CDF figure (now Fig 2a and 2b) by dividing into two panels (whites/people of color and poverty/not in poverty) and adding a curve representing the difference between each CDF pair. We have labeled the maximum CDF % difference as well as the distance corresponding to the maximum CDF difference.

We have also removed the illustrative example text using a four-mile distance, as that caused confusion among reviewers.

**For the comparison of EJ- and Non-EJ-communities, I would recommend using some systematic analysis (i.e., ANOVA) to evaluate if the transitions were significantly different as stated in Lines 392-393.**

We thank the reviewer for this suggestion. We conducted ANOVA of changes from 1995-2015 in the total number of EGUs by fuel type in EJCs vs non-EJCs. We have added the following sentences to the Results section (lines 364-370 in this resubmission) and include a supplementary table:

*To assess the statistical significance of changes in the number of power plants, we conducted one-way ANOVA of the frequency of oil-, natural gas-, and coal-fired power plants. We excluded biomass-fired plants because no biomass plants regulated by RGGI were sited in EJ communities in our analysis; we also excluded plants that did not report fuel type. p-values indicate that the number of power plants of each fuel type are different in statistically-significant ways in EJCs compared to non-EJCs (Supplemental Table 1).*

**lastly, while the study offers a good discussion and details on limitations, there is still a lack of focus on what are the implications of the results and how the environmental planners along with community stakeholders might benefit from the findings of this study. Hence, it is recommended to discuss the potential implications of the findings from the perspective of decisions and policy.**

This has been raised by other reviewers as well and we have added more discussion as requested to our discussion and conclusions sections.

**Reviewer #3: This study addresses a vital question: whether market-based greenhouse gas emission reduction mechanisms such as RGGI increase environmental injustice. But it suffers from a fatal flaw in methodology and also from a lack of effort to explain the patterning of findings.**

In response to related comments about the reasons for the patterns we found, we have now noted in the conclusions that there are multiple factors that could explain the differential patterns we found between EJ and non-EJ communities. These factors are both endogenous (e.g., RGGI incentives and allowance pricing), and exogenous (e.g., declining natural gas prices, Big Green campaigns) to RGGI. Our research design does not allow us to probe into the very likely dynamic interplay of these factors, and thus we have left the answer to this question to future research.

Relatedly, we have noted in our limitations section that our analysis and data did not allow us to establish if the steeper increase in natural gas units in environmental justice EJ communities occurred due to conversion of coal units to gas, or if it entailed the construction of new plants.

**The authors selected on the dependent variable (by only including the 285 Census tracts hosting one EGU, rather than all Census tracts in the 11 RGGI states). This has biased the analysis, which only compares host communities with host communities; non-host communities (that tend to be whiter and higher in income) are entirely left out of the analysis which results in under-measuring environmental inequality (see Mohai 1995, his famous critique of Anderton, Anderson, Oakes and Fraser 1994).Mohai, Paul. "The demographics of dumping revisited: examining the impact of alternate methodologies in environmental justice research." Virginia Environmental Law Journal (1995): 615-653.**

We appreciate the distinction that Reviewer 3 has made in regards to potential differences in estimating environmental inequities due to the choice of host-to-host vs host/non-host comparisons. We agree with Reviewer 3 that by restricting our analysis to census tracts hosting power plants we are underestimating the true burden of exposure from power plant pollutants that extends to other communities outside host communities. However, assessing the true burden of exposure from power plant pollutants requires atmospheric chemistry and dispersion modeling, which is outside the scope of our research and which we have alluded to in the limitations section. Because our analysis is focused on emissions, frequency, generation, and capacity factors of power plants hosted in RGGI communities, an analysis of host/non-host tracts in our research is not feasible.

We have probed further into Reviewer 3’s comment, however, and have assessed the demographics of host/non-host census tracts in the RGGI states in our study to evaluate if in fact communities not hosting power plants in RGGI are whiter and higher in income. We find that on average, the 219 host tracts are 12.6% in poverty and 29.7% people of color, while the 11,725 non-host tracts are on average 12.8% in poverty and 39.0% people of color. We have assessed the statistical significance of these averages and found that the very similar means of percent in poverty between host and non-host tracts are not different in statistically-significant ways (p-value = 0.3571), while the small p-value for the difference of means of percent people of color for host vs. non-host tracts (p-value= 4.616e-05) suggests these differences are statistically significant. This is by no means a complete assessment of power plant-related environmental inequalities between host and non-host communities; as it is outside of the scope of our present work, we respectfully decline to include a host/non-host analysis in our manuscript. In lines 504-507, we have added the argument in Mohai et al (1995) as a limitation of our research, and in the conclusions in lines 593-597 we assert the contributions of our host community analysis to advancing equitable emissions reductions in RGGI.

**The authors demonstrate a good working knowledge of environmental justice issues; if they choose to redo the analysis to eliminate bias, more could be done to increase this study’s contribution to the scientific literature. Specifically, the paper suffers due to lack of explanation of the findings: what is driving the shift? The authors would need to at least try to explain the patterning of environmental inequalities found so that the paper can be stronger scientifically and also be a better contribution for crafting policy.
Due to the methodological problems, I am recommending that the paper be rejected. But I include detailed comments below in case the authors decide to re-do the analysis:
Comments on Introduction and Literature Review:
1. There needs to be some explanation of why RGGI matters for environmental inequality: how is RGGI hypothesized to decrease or increase environmental burdens for EJCs? How, if at all, might RGGI change the market calculation of what type of power plant might be profitable? The authors cite a few papers on these questions but I know there are many more.**

We agree with the reviewer that there needs to be a robust discussion of the importance of RGGI for environmental quality. In regards to this, we have already discussed the concerns about distribution of benefits from EJ scholars and advocates in the literature review. We now reinforce these points in our conclusions. To directly answer the reviewer’s question, we have stated that RGGI matters for environmental quality because power plants in RGGI exist in a managed policy landscape and that, moving forward, there need to be policies that bring benefits to overburdened communities (starting on line 592).

The economics of power plants and the factors that could make some plants more profitable than others is outside of our area of expertise; furthermore, since our study is not an economic analysis of those factors, that question is outside of the scope of our study. What we have noted, however, is the concern among EJ scholars for “the potential for carbon market forces to drive increases in carbon and co-pollutant emissions as utilities pursue lower-cost emissions reductions” (lines 582-585), and we think this must be balanced against non-RGGI explanations for emissions reductions, e.g., Kim and Lee’s finding that availability of natural gas and not necessarily market incentives have driven emission reductions in RGGI.

**Comments on data and variables:
1. It was not clear why “prime movers” mattered for this analysis. Do they have an effect on emissions? If so, how? If prime mover is just another way of referring to fuel, the authors should omit that phrase.**

We agree with the reviewer that the term “prime mover” is not critical for our analysis. We included it as background information to understand power plant data aggregation. We have removed Figure 2 in our original submission as well as the prime mover term from our manuscript (and renumbered our figures).

**2. On Page 10-11, the authors don’t provide a clear definition of “environmental justice community.” Does this mean census tracts above a certain threshold of percent people of color AND above a certain threshold of people in poverty (or was it OR a certain threshold of people in poverty)?**

Reviewer 2 has raised a similar issue regarding the definition of EJ communities in our study. See our reply above.

**And what were “non-EJC outliers?”**

As we have stated in lines 330-332, non-EJC outliers are “five tracts with percent people of color one standard deviation above the mean [that] fell in the non-EJC category and are labeled as non-EJC outliers (Fig 3).”

**3. On page 21, describing the limitations of the study, the authors state that it would have been preferable to consider people living under the poverty level (presumably, for a comparison with those living at poverty level), but due to limitations in Census data this was not possible. Given that the poverty level is such an outmoded undercount of people living in poverty, I doubt that comparing people in poverty with people in deep poverty would have been as useful as comparing people in poverty with those better off. But the authors could have compared people at 100% of poverty level with people at 200% of poverty level (the near-poor). These data are available in Table POV01, which should be available for the years up to 2014.**

We agree with the reviewer that the poverty level is an outmoded undercount of people living in poverty. We also agree that comparing people in poverty with people in deep poverty would not be useful, but we are not saying that would have been preferable. In lines 534-536, we are saying that it would have been preferable to combine the poverty level of people living under the poverty level with that of those living at the poverty level. We have modified the sentence to make this clearer, as the sentence may not have communicated that clearly.

We believe our clarification and modification of lines 534-536 makes this a moot point, but we would like to clarify that the Census defines “near poor” differently from what the reviewer has noted. The US Census Bureau defines “near poor” as the population “with family income between 100 and 125 percent of the poverty thresholds” (see <https://www2.census.gov/library/publications/2014/demographics/p60-248.pdf>).

**Comments on methods:
1. Unfortunately, the methods used in the analysis were fatally flawed: the authors selected on the dependent variable by only including the 285 census tracts that host EGUs in the analysis. This has biased the analysis because it compares host communities with host communities, leaving non-host communities entirely out of the analysis. The scope of the analysis is very important, as this methodological choice has been proven to underestimate environmental inequality (see Mohai 1995).**

The reviewer has made this point above and we have addressed it.

**Aside from this serious problem, other methodological choices were not adequately explained:
2. It is not clear why the distance of four miles was chosen to represent proximity to EGUs. Is there some scientific justification for defining proximity in terms of four miles from the centroid of each census tract, rather than one mile, two miles, etc.?**

Reviewer 2 has made a similar point where using four miles as an example to illustrate differences at that distance caused confusion. See above for our modifications to the language and figures to better communicate our meaning.

**3. The authors seemed to miss out on some of the precision with which distance-based measures can find environmental inequalities. Did people of color or people in poverty live closer to EGUs than others? Or was a four-mile radius the basis for the analysis?**

Again, Reviewer 2 has made a similar point where using four miles as an example to illustrate differences at that distance caused confusion. See above for our modifications to the language and figures (now Figs 2a and 2b) to better communicate our meaning.

**Comments on interpretation of findings:
1. The authors should at least try to find explanations for these patterns, such as the finding that there were fewer coal-fired EGUs and more natural gas-fueled EGUs in EJ communities. Below are some possible explanations that could be explored:**

We agree with the reviewer that explaining the reasons for the pattern in our findings is important. We have done that in a couple of places in our discussion. In the limitations section we have noted that the data used did not did not allow us to establish if the steeper increase in natural gas units in environmental justice EJ communities occurred due to conversion of coal units to gas, or if it entailed the construction of new plants. We have also noted that:

*The coal to natural gas transition has occurred in the context of a largely successful effort from environmental, clean and renewable energy, and climate change mitigation advocacy groups to retire coal-burning power plants to reduce GHG emissions and enable the transition to renewable energy. In our analysis, the disaggregate benefits of that transition—as measured by inequities in number of units and generation of electricity from natural gas— appear to have gone to non-environmental justice communities.*

**• Natural-gas power plants may be newer than coal-fired power plants, and thus may have been sited recently. Since 1990 there has been more opposition to siting environmentally hazardous facilities; non-EJ communities may have successfully blocked new power plants, leaving EJ communities “the path of least resistance.”**

We agree with the reviewer’s speculation that it is likely that non-EJ communities have succesfully blocked new power plants. We also think that may be a result of climate and energy advocacy groups’ efforts having aligned more with those communities (read: Whiter and wealthier). However, we do not have evidence to back up this potential explanation and thus we have added that as a question for future research.

**• This also could be driven by zoning: it is well established that minorities and the poor are more numerous in places zoned for industrial use.**

We agree. Starting on line 614, we have already mentioned the likely role of discriminatory zoning and land-use planning decisions on the pattern of inequities that we found, and we have now added Mohai and Saha (2015) as more evidence:

*These are critical questions to answer in light of inequities in enforcement of environmental, civil rights, and public health laws and policies, as well as pervasive discriminatory zoning and land-use practices, flaws in industrial risk assessment, and exclusionary practices that limit meaningful participation of environmental justice communities in decision-making, all of which prompted the emergence of the environmental justice in the first place [65,66].*

**• Investment in new (natural gas) power plants or in the conversion of outmoded ones may have occurred in more densely populated urban and metropolitan areas versus rural areas. Populations in rural areas tend to be whiter and poorer.**

We agree this is a possible explanation for the inequitable coal-to-gas transition in RGGI. Assessing these possible differences is outside of the scope of our research, and we have noted it as an area of future research.

**• It is likely that there is a lot of variation in the patterns of proximity to EGUs in RGGI-participating states: states that are white, rural and poor with little investment in new infrastructure (e.g., Maine) may be the same states with more outmoded coal-fired plants; states that are urban and diverse with extremes of wealth and poverty (e.g., New Jersey) may have more natural gas plants.**

To explore this possible explanation for the inequitable pattern in the coal-to-gas transition, we calculated cumulative density functions (CDFs) for individual states in RGGI. Below we show CDFs for NY, NJ, VT, and ME, three states with EJ-relevant demographics differences, as the reviewer states. Although there are slight differences between more urban and diverse states with more extremes of wealth and poverty (e.g., NY and NJ) and more rural, whiter states (e.g., ME and VT), the general pattern of larger fractions of EJ populations than non-EJ populations living in proximity to power plants is very similar. In our analysis we did not calculate population proximity separately for coal and gas-operated power plants, so we cannot comment on state-level differences in specific fuel types. Thus, we have no empirical basis to speculate in our discussion that the differences that the reviewer is mentioning can explain the inequitable patterns in the coal-to-gas transition.

**• If any of these explanations are correct, they might explain the finding that CO2 and SO2 emissions in EJCs were higher between 1995-2005 but reductions were larger in EJCs after 2007.**

In our analysis, we have visualized longitudinal patterns of emissions for a variety of fuel types for a 20-year period (1995-2015), and in our discussion we have noted visual patterns of changes in emissions trajectories. We have not modeled the factors that may account for changes from year to year. While we do think exploring the factors that could influence emissions changes from year to year in RGGI power plants is an interesting research question, that is outside the scope of our analysis and it would require more data than was available for this study.

**2. The finding regarding emissions appears to be a bright spot: emissions have gotten lower since 2005 for EJCs. But the authors needs to do more to illuminate an understanding of this finding: did the pattern of emissions change due to the shift from gas to coal? Was it due to plant modernization (better pollution controls, cleaner fuels, or both)? Was it because methane emissions increased while CO2 and SO2 decreased? All of these questions are really crucial ones for redressing environmental inequalities.**

Note - we believe the reviewer means “shift from coal to gas” (not “gas to coal”). Visually, our CO2, SO2, and NOx emissions graphs suggest that reductions in coal emissions drove observed reductions.

We agree that understanding the reasons for changes in emissions is important for redressing environmental inequities. As we have said in our reply to Reviewer 3’s point #1 above, elucidating the reasons exogenous or endogenous to RGGI for changes in emissions trajectories is outside of the scope of our study. Similarly, we are not experts in power plant pollution control technology, so we are not able to assess their role in reducing emissions. Finally, we did not have access to methane emissions data, so we cannot speculate on the relationship between CO2/SO2 emissions in RRGI power plants and methane. We have, however, added a question for future research on the role of pollution control technologies in our conclusions.

**3. Finally, what is the significance of the timing of the shift? Could it be due to policy (Energy Policy Act, Clean Power Plan, etc.)? Or the economy (gas prices)? If finding answers is beyond the scope of this study, the authors could speculate on these questions and suggest future research.**

In response to similar comments, we have stated that multiple factors both exogenous and endogenous to RGGI could be the drivers of the patterns we found. We have modified a sentence from our first submission to leave this more generally as a question for future research in lines 608-610:

*But which policy, market, or other forces can account for the faster ramp up of natural gas power plants in environmental justice communities?*

**Comments on Conclusions:
1. The authors should mention the impacts on EJCs from the expansion of shale gas and oil, including fracking wells, oil trains, gas pipelines, liquid natural gas export terminals and compressor stations, among others.**

We have added mention of these sources of EJ inequities in lines 507-510.

**Comments on Figures:
Figure 2: I was unable to understand from the manuscript why “prime mover type” was important. If it was because it affects the amount or toxicity of emissions, this should be made clear. I am not sure what this figure adds; if space limitations become an issue, I recommend omitting it.**

In response to a similar comment above, we have removed the term “prime mover” and the power plant/EGU schematic (Fig 1 in first submission).

**Figure 3: what are the “outliers”?**

As we have stated in lines 330-331, non-EJC outliers are “five tracts with percent people of color one standard deviation above the mean [that] fell in the non-EJC category and are labeled as non-EJC outliers (Fig 4).”

**Figures 5 – 10: There are too many figures, which take up space the authors need.**

We have removed the electricity-generating unit schematic (Fig 2 in original submission). We deem the number of figures necessary to communicate our findings.

**Mohai, Paul. "The demographics of dumping revisited: examining the impact of alternate methodologies in environmental justice research." Virginia Environmental Law Journal (1995): 615-653.**

**Reviewer #4:** **Title: Exploratory analysis of changing power plant pollutant burdens on environmental justice communities in the Regional Greenhouse Gas Initiative states.**

**Summary of paper: While there have been many studies that evaluate the spatial distribution of environmental hazards to environmental justice populations (predominantly characterized as low income communities and communities of color), few studies have evaluated environmental justice burdens in GHG emissions markets. This study contributes to environmental justice literature by assessing pollution and power plant disparities in RGGI states. Authors found that a larger share of environmental justice populations live near power plants than non-ej populations and that the coal-to-natural gas transition was more evident in environmental justice communities. Reductions in CO2, NOx, and SO2 emissions were found to be similar in EJ communities and non ej communities over the time period studied, with a few differences.

This paper offers valuable contributions to the field and contextualizes the importance of these considerations for GHG emissions and broader climate policy while discussing limitations clearly. However, there are a few areas with needed clarification of methods, presentation and discussion of certain results, citations, and supporting figures.

Major changes:
Literature Review (e.g., Line 86): add/incorporate citation - McCoy E. Which came first, coal-fired power plants or communities of color? Assessing the disparate siting hypothesis of environmental justice (Doctoral dissertation).**

**Literature Review: add / incorporate citation - Diana, Ash, and Boyce (2021) Green for All: Integrating Air Quality and Environmental Justice into the Clean Energy Transition -- <https://peri.umass.edu/economists/michael-ash/item/1408-green-for-all-integrating-air-quality-and-environmental-justice-into-the-clean-energy-transition>**

We have incorporated McCoy 2017 and Diana, Ash, and Boyce (2021) in our literature review.

**Lines 189-192: In this paragraph, there should be more clarification on the exact variables that were used from the Census to form the “percent people of color” and white variables in the analysis. Specifically, was non-Hispanic white used to identify white populations in the analysis? Was the “percent people of color” variable built so that certain minority populations were not double counted? i.e. if the race variables of the Census (African American alone, Asian alone, etc) were combined with the ethnicity variable, ALL who identify as Hispanic or Latino of any race, then people who identify as both African American AND Hispanic or Latino would be double counted in the population of color. The same would be true for Hispanic White individuals in both the white population and the people of color population. Further clarification is needed to confirm this error did not take place. If populations were double counted, this could significantly impact the analysis and require major revisions.**

We thank the reviewer for this question. We overlooked specifying that we used the US Census counts for non-Hispanic African Americans (B03002e4), non-Hispanic American Indians or Alaska Natives (B03002e5), non-Hispanic Asians (B03002e6), Hawaiians/Pacific Islanders (B03002e7), and other non-Hispanic persons of a non-White race (B03002e8) in our analysis (see <https://www2.census.gov/geo/tiger/TIGER_DP/2013ACS/Metadata/BG_Metadata_2013.txt> for complete metadata descriptions of the US Census variables used). To answer the reviewer’s question, “percent people of color” was built from these variables, ensuring that no persons of color were overcounted.

**Lines 199 - 205: A figure depicting the methods of CDF would clarify the process followed for the reader**

The application of CDFs in assessing environmental inequities is not a novel method, having been advanced by Zandbergen and Chakraborty (2006), which we have cited in lines 199-205. While we realize the CDF method may not be straightforward for all readers, other reviewers have pointed out that the number of figures in the manuscript is large and have requested reducing the number figures. Therefore, we respectfully decline to add a figure to explain the CDF method. However, we take Reviewer 4’s concern seriously and have modified the language to provide a clearer explanation of the CDF starting in lines 217 (*Population proximity to power plants subsection*).

**Line 242: The analysis drops to focus only on the 285 Census tracts that contain an EGU. It would be helpful to clarify how many total ej and non-ej tracts there were regardless of presence of an EGU in the study area and it seems as though some results are presented in a way that makes the distinction between presence of EGU or not unclear. It would aid the analysis to consider the rate of EGU in non-ej communities relative to ALL non-ej communities and the rate of EGU in ej communities relative to ALL ej communities.**

A similar point related to the inclusion of tracts not hosting EGUs has been brought up by another reviewer. As we have said above, our analysis is focused on assessing differences in power plant characteristics between EJ and non-EJ census tracts hosting plants, and is not focused on assessing the EJ/non-EJ status of all communities in RGGI. However, we used all 11,813 Census Tracts in RGGI states in the k-means cluster analysis in order to obtain a classification of Census Tracts that is representative of the distribution of poverty and race/ethnicity across RGGI states. We realize that we have not adequately conveyed that we created the EJC/non-EJC classification based on all Census Tracts in RGGI but subsequently selected only EGU host tracts, and that this has created confusion among reviewers. We have made this clear in lines 274-278. We also have taken into consideration the reviewer’s desire to see the EJC/non-EJC classification for all tracts, and in line 304 we have referenced a supplementary figure showing the classification for all tracts.

**Lines 232 - 246: it’s not clear what the threshold for defining an EJ community is and if there is no threshold, what makes an ej community distinct from a non-ej community.**

Reviewer 2 has raised similar concerns about the lack of clarity on our description of how we identified EJ vs. non-EJ communities. We have modified the language on lines 260-270 to more clearly explain our method.

**Lines 291 - 298: the presentation of the results on the number of EGUs cited in ej and non ej tracts is not convincing. One possible clarification would be including the rate of EGUs in EJ or non EJ tracts relative to ALL ej or non EJ tracts in the study area, not just those with EGUs.**

In lines 291-298, we are trying to make two points. First, that most EGUs are sited in non-EJCs, as most Census Tracts hosting EGUs were classified as non-EJCs. Second, that the relative frequency of multiple EGUs in any one Census Tract is higher in EJCs than in non-EJCs. In the discussion section we suggest that this implies a higher burden of power plant pollutant exposure among EJCs than non-EJCs (a finding similar to that of Cushing et al’s EJ assessment of the CA cap and trade program). In that regard, presenting the rate of EGUs in EJCs and non-EJCs relative to all EJCs/non-EJCs across RGGI is not useful to make these points. Furthermore, the scope of our research is assessing differences in power plant characteristics between EJ and non-EJ census tracts hosting plants, and is not focused on assessing the EJ/non-EJ status of all communities in RGGI.

**Continued below:**

**Lines 412 - 413: given the results, the statement “environmental justice communities more frequently host multiple EGUs compared to non-environmental justice communities” seems inaccurate because non-ej communities still host a higher number of EGU units (49) than ej communities (20). Reading the data presented, a reader could interpret that a larger proportion of EGUs are in non-ej communities, both when sited as 1 unit or 2-5 units together -- this seems to be different from how this data is discussed by the authors. This result (above) and discussion of the result here should be removed, reworded/clarified, or considered from another perspective (i.e., as mentioned above, percentage of ej community tracts with EGUs / ej community tracts total vs percentage of non-ej community tracts with EGUs / non-ej community tracts total) in order to be supported by the data.**

We agree with the reviewer that stating that “environmental justice communities more frequently host multiple EGUs compared to non-environmental justice communities” is inaccurate. In lines 446-447, we have corrected that sentence to read:

*…the relative frequency of multiple EGUs in any one Census Tract is higher in environmental justice communities than in non-environmental justice communities.*

Similarly, in the Results section in line 347, we have changed the sentence to convey that the differences in siting of multiple EGUs in any one community is relative with respect to all census tracts in each of the EJC and non-EJC categories:

*When looking at the distribution of EGUs within communities, we find that EJ communities have a higher* ***relative frequency*** *of multiple EGUs sited within when compared to non-EJ communities…*

We understand the reviewer’s suggestion that we compare siting frequency to all census tracts regardless of host/non-host status, but we respectfully decline to do so. This is in line with our focus on EGU host tracts throughout the paper. However, we believe we have clarified the meaning of our findings with these modifications.

**Minor changes:
Line 45: missing citation**

We have added the missing citation.

**Lines 88-90: cut, repeated verbatim in lines 91-93**

We have removed the repeated lines.

**Line 102: missing citation**

We have added the missing citation.

**Line 170: clarify the analysis is limited to plants greater than 25 MW. In the discussion, it is stated that above 25 MW are those that fall within the scope of RGGI, so it would be helpful to the reader if that was stated earlier in this spot as well.**

In lines 187-188 we have stated that we have focused our analysis on RGGI plants with 25MW or larger capacity.

**Line 232 - remove word unbias. Any method requires choices that include bias.**

We have removed the word "unbiased” and rewritten the sentence.

Other notes

1. We have corrected an incorrect number of census tracts starting in line 254. The number was incorrectly listed as 285, but has been corrected to 211, which matches the total number of census tracts on Figure 4.
2. Some reviewers stated that we did not provide access to our study’s underlying data and methods. However, in the Additional Information section of the submission interface, we specified that the data are available at <https://osf.io/tu2hj>. We have now made the repository public for their review, and we have indicated to the editors that upon acceptance for publication, we will move the data to a public repository that can issue a DOI and make the data available.
3. We have added the longitudinal analysis of CO2, NOx, and SO2 emissions explicitly as a research question in the introduction.

References

Gibergans Bàguena, José, Carme Hervada Sala, and Eusebio Jarauta Bragulat. "The quality of urban air in Barcelona: a new approach applying compositional data analysis methods." Emerging Science Journal 4, no. 2 (2020): 113-121.

Okeke, Francis O., Ibem O. Eziyi, Clifford A. Udeh, and Emmanuel C. Ezema. "City as Habitat: Assembling the fragile city." Civil engineering Journal 6, no. 6 (2020): 1143-1154.

Angelevska, Beti, Vaska Atanasova, and Igor Andreevski. "Urban air quality guidance based on measures categorization in road transport." Civil Engineering Journal 7, no. 2 (2021): 253-267.
