# Supplementary material for: Environmental justice and power plant emissions in the Regional Greenhouse Gas Initiative states
Source: PLoS One. 2022 Jul 20;17(7):e0271026. doi: 10.1371/journal.pone.0271026 (PMC9299318; doi:10.1371/journal.pone.0271026)
Supplement: Attachment — Submitted filename: letter from reviewer.docx [file pone.0271026.s005.docx]

**Reponse to reviewers**

Thank you for the comments that the reviewer has provided to our manuscript. We have addressed each comment in our response below in **bold**.

==============================

I have served as a reviewer for the first draft of this manuscript.

The article is mostly ready for publication, but minor revisions are needed because the authors’ interpretation of their findings in the Findings, Discussion and Conclusions sections are not consistent with the data presented in the article (see Comments below). Revisions on interpretation of findings are necessary for publication as is clarifying the discussion of findings on Page 18. The recommendation that the authors emphasize the disparity in the number of EGUs is suggested but not required.

Comments on data and variables:

- In the revised version of the paper, the authors are no longer testing for disparities in the presence/absence of EGUs within a 4-mile radius, but rather testing for disparities in distance/proximity to EGUs. They are still comparing conditions only in host communities, but seeking to determine whether, within host communities, EJCs live closer to EGUs than non-EJCs. The methodology used (CDF function) was appropriate to the research question, and the researchers were able to provide convincing evidence of disparities in proximity.

Comments on guiding questions and their relationship to findings and interpretation of findings (Page 7):

Question 2 asks if income and race are an appropriate basis for delineating EJ communities from non-EJ communities.

Question 3 asks what is the distribution of the polluting potential as measured by fuel type, total net generation, capacity factor and actual pollution from CO2, SO2 and NOx in EJCs vs. non-EJCs.

Findings:

1. People of color are 23.5% more likely to live within 0-6.2 miles of an EGU; people living in poverty are 15.3% more likely to live within 0-4.9 miles of an EGU.

2. 42.6% of EJCs host 2-5 EGUs but only 28% of non-EJCs do.

- This is a rather significant point, one that should be emphasized more when discussing possible disparities in exposures to air pollutants from EGUs.

**We have added this finding to our discussion in lines 422-424 and contextualized what it may mean for higher exposure burdens in lines 432-433.**

3. Overall, from 1995-2015 % of coal-fired EGUs smaller in EJCs (higher in non-EJCs).

4. Since 2003: % of natural gas EGUs higher in EJCs than non-EJCs.

5. Total net generation in non-EJCs about 3 times as high as in EJCs (stated on Page 17).

- Findings 3 and 5 provide another indication that RGGI has not affected all communities evenly, since non-EJCs face higher risk of exposure to pollutants from coal-fired power plants than EJCs, and pollution potential in terms of generation is higher for non-EJCs. It is higher in other ways for EJCs (EJCs closer to EGUs, more EJCs host multiple EGUs, EJCs suffer more potential exposure from gas-fired plants). The authors should mention and discuss all the ways in which environmental inequalities manifest.

**We agree that non-EJCs face higher exposure to coal-fired power plants than EJCs, as the percentage of coal-fired power plants in EJCs is lower than that in EJCs. We do not agree that the same is true of generation. We have reported total net generation, which is expected to be higher in non-EJCs because the total number of plants is higher in non-EJCs (indicated by the numbers along the x-axis on e.g, Fig 5). Furthermore, the trend of generation in EJCs has been increasing, while in non-EJCs generation increased until 2005, then dropped afterwards (black line in Fig 5). We do agree that environmental inequities manifest in many varied ways, but we do not think that the higher frequency of coal plants in non-EJCs is an inequity on equal footing to the higher frequency of natural gas plants in non-EJCs. While the relative frequency of coal in each type of community appears, at least visually in Fig 5, to have remained relatively stable during 1995-2015, the natural gas ramp up did not, and as we have pointed out as one of our main findings, gas ramped up faster in EJCs.**

- This implies that non-EJCs have benefited less from the shift from coal to natural gas than EJCs. This, too, should be mentioned and discussed.

**We disagree with this interpretation. The percent of coal power plants in each type of community has remained fairly constant, with the exception of EJCs after 2012, when it appears to reduce slightly (Fig 4). Again, we point to one of our main findings that natural gas generation and total number of natural gas units ramped up faster in EJCs, which indicates that RGGI has not explicitly considered potential impacts or inequitable burdens on environmental justice communities.**

- These results also imply that the definition of EJCs in purely demographic terms has some important limitations: it has the effect of making distributional inequalities that affect communities not defined as EJCs invisible, perhaps because their demographics are just slightly too white or not poor enough. The authors should at least mention this, perhaps couching it in terms of answering Question 2.

**We agree with this limitation.**

6. Between 1995-2005, CO2 emissions in EJCs were slightly higher; reductions were larger in EJCs after 2009.

- This implies that EJCs might have benefited somewhat, or in some ways, from reductions in CO2 emissions due to RGGI. While there may be other factors that partially or totally eclipse this benefit, the authors should not ignore this finding.

**We have added this finding to our discussion of the multiple ways in which environmental inequities manifest.**

7. After 2003, CO2 emissions from natural gas rose in EJCs more than in non-EJCs.

**We have modified lines 580-582 to include this.**

8. (Page 18): S02 emissions higher in both types of communities (or just EJCs)?

- The authors should clarify the discussion on Page 18; as written, it is unclear.

**We have corrected the sentence to state that SO2 emissions in 1997 and 1999 were higher in EJCs (lines 391).**

9. Starting in 2003, S02 emissions decreased in EGUs in both types of communities but reductions were larger in EJCs especially after 2007.

- As is true of #6, the authors need to discuss this finding and put it into context.

10. S02 emissions mostly from coal and oil; decreased more in EJCs after 2009.

- Same as #6 and #9.

**We have added language in the discussion to highlight this.**

11. Average NOX emissions were larger in EJCs during 1995-2005 but dropped markedly after 2000.

- Same as #6, #9 and #10.

**We have added language in the discussion to highlight this.**
